# Genome-scale metabolic models of *Microbacterium* species isolated from a high altitude desert environment

**DOI:** 10.1038/s41598-020-62130-8

**Published:** 2020-03-27

**Authors:** Dinka Mandakovic, Ángela Cintolesi, Jonathan Maldonado, Sebastián N. Mendoza, Méziane Aïte, Alexis Gaete, Francisco Saitua, Miguel Allende, Verónica Cambiazo, Anne Siegel, Alejandro Maass, Mauricio González, Mauricio Latorre

**Affiliations:** 1 Laboratorio de Bioinformática y Expresión Génica, INTA-Universidad de Chile, El Líbano, 5524 Santiago, Chile; 2 Fondap Center for Genome Regulation (CGR), Avenida Blanco Encalada, 2085 Santiago, Chile; 3 Mathomics, Center for Mathematical Modeling, Universidad de Chile, Santiago, Chile; 4 Systems Biology Lab, Amsterdam Institute for Molecules, Medicines and Systems, VU Amsterdam, De Boelelaan 1108, 1081 HV Amsterdam, The Netherlands; 5 IRISA, UMR 6074, CNRS, Rennes, France; 6 INRIA, Dyliss Team, Centre Rennes-Bretagne-Atlantique, Rennes, France; 7 Department of Chemical and Bioprocess Engineering, School of Engineering, Pontificia Universidad Católica de Chile, Avenida Vicuña Mackenna, 4860 Santiago, Chile; 8 Department of Mathematical Engineering, Universidad de Chile, Santiago, Chile; 9 Instituto de Ciencias de la Ingeniería, Universidad de O’Higgins, Av. Viel 1497, Rancagua, Chile

**Keywords:** Soil microbiology, Metabolic engineering

## Abstract

The Atacama Desert is the most arid desert on Earth, focus of important research activities related to microbial biodiversity studies. In this context, metabolic characterization of arid soil bacteria is crucial to understand their survival strategies under extreme environmental stress. We investigated whether strain-specific features of two *Microbacterium* species were involved in the metabolic ability to tolerate/adapt to local variations within an extreme desert environment. Using an integrative systems biology approach we have carried out construction and comparison of genome-scale metabolic models (GEMs) of two *Microbacterium* sp., CGR1 and CGR2, previously isolated from physicochemically contrasting soil sites in the Atacama Desert. Despite CGR1 and CGR2 belong to different phylogenetic clades, metabolic pathways and attributes are highly conserved in both strains. However, comparison of the GEMs showed significant differences in the connectivity of specific metabolites related to pH tolerance and CO_2_ production. The latter is most likely required to handle acidic stress through decarboxylation reactions. We observed greater GEM connectivity within *Microbacterium* sp. CGR1 compared to CGR2, which is correlated with the capacity of CGR1 to tolerate a wider pH tolerance range. Both metabolic models predict the synthesis of pigment metabolites (β-carotene), observation validated by HPLC experiments. Our study provides a valuable resource to further investigate global metabolic adaptations of bacterial species to grow in soils with different abiotic factors within an extreme environment.

## Introduction

In recent years, there has been increased interest in the study of *Actinobacteria* from desert areas. As noted by previous researchers, this class possess bacteria with a variety of attractive metabolic qualities that make them good candidates to survive in desert areas^1^, including their extensive metabolic capability to degrade and utilize compounds from nutrient poor environments and their ability to synthesize secondary metabolites and natural antibiotics^2^. In fact, *Actinobacteria* is the dominant class present in desert locations^3^, and their ability to adapt to this kind of environment is the focus of intense research. For example, Lynch and collaborators found that over 98% of the lineages present in the vicinity of a volcano in the Atacama Desert corresponded to *Actinobacteria*, and that their metabolisms were adapted to utilize atmospheric gases present in this ecosystem^4^. One genus that belongs to the class *Actinobacteria* is *Microbacterium*, which is present in a vast variety of environments, including desert soils, marine sediments, and the human gut.

Recently, *Microbacterium* sp. CGR1 was isolated from the Andes mountains in the central Atacama Desert^5^. Its complete genome sequence revealed the presence of putative components related to pH and salinity tolerance, representing the first genome of the genus *Microbacterium* sequenced and assembled in a single contig. In addition, two other strains of *Microbacterium* (*Microbacterium album* and *Microbacterium deserti*) were isolated and sequenced from desert soils from Saudi Arabia. Biochemical assays highlighted important differences among them in their basal metabolism, mainly related to lipid and carbohydrate (sugar) compositions^6^. Recently, a second *Microbacterium* was obtained from the Atacama Desert under a salt crust in the shore of the saline Lejía Lake^7^. Lejía Lake, which is nested at the base of Lascar Volcano, is one of the highland lakes at risk of disappearing due to climatic change. Despite the extreme conditions present in this lake, a diverse group of bacteria have been found to reside in this environment^7,8^. One of these bacteria is *Microbacterium* sp. CGR2, which has high pH (up to pH 12) and salinity (5% NaCl) tolerance levels^7^.

Research to date demonstrates that *Microbacterium* from desert soils display interesting and specific features in terms of the configuration of their global metabolism, making it necessary to identify and characterize the complete set of chemical reactions that occur in *Microbacterium* species in order understand how they survive under extreme dry environments.

In this context, a valuable tool for the systematic study of microbial metabolism of new species is the genome scale model (GEM)^9^. GEMs are networks of biochemical reactions of a given microorganism that encompass important metabolic properties of cellular biochemical networks, such as mass and redox balance, and energy requirements^10^. By using information obtained from annotated genomes and introducing some assumptions, models are capable of: (1) interpreting current biochemical information for metabolic and genetic context, (2) predicting metabolic capabilities, and (3) guiding future experiments to increase the knowledge of the metabolic capabilities of a given organism^11^. In recent years, several manually curated GEMs have been reconstructed to explore the capabilities of different microorganisms^12,13^ with an emphasis on model organisms, such as *E. coli*^14^ or *Bacillus subtilis*^15^, but also include several species of *Actinomycetes*^16^. However, to date there are no manually curated GEMs for organisms from the genus *Microbacterium* and there is only one GEM of a bacteria from the Atacama Desert^17^.

In order to generate important information about global and specific metabolic pathways, which can be correlated with the ability of bacteria to survive given the local context of an extreme environment, we reconstructed and characterized the first GEMs of *Microbacterium* species. Our analysis shows that the capacity to survive under extreme conditions correlates with the presence of a complete set of metabolic pathways related to the production of osmolytes and the connectivity level of particular metabolites. In addition, *Microbacterium* models predicted the synthesis of pigment molecules, while an HPLC assay determined the presence of β-carotene, a strongly colored red-orange pigment used in biotechnological applications.

## Material and Methods

### *Microbacterium* sp. CGR1 and CGR2 growth conditions

CGR1 and CGR2 were previously isolated from the top layer (10 cm depth) of soil samples obtained from the slope of Lascar Volcano (23°30′S and 67°42′W and 4480 m a.s.l^5^; and near Lejía Lake (23°29′S and 67°41′W and 4327 m a.s.l^7^;, respectively Both strains were grown in tryptic soy agar (TSA) or tryptic soy broth (TSB)^18^.

### Soils condition tolerance assays and elemental content

pH and temperature of the Lascar Volcano and Lejía Lake soil samples were determined *in situ* using a pH meter (Hanna Instruments, Woonsocket, RI) and an electronic thermometer (Orion model 290), respectively. Electrical conductivity (EC) was determined in a 1:5 soil/water extract. Total elemental profiles from soluble soil extracts were determinate by Total Reflection X-ray Fluorescence (TXRF)^19^.

For soil condition tolerance assays, bacterial growth curves were determined. In order to emulate the specific soil conditions from which CGR1 and CGR2 were isolated, 10 g of soil from Lascar Volcano (Volcano) and Lejía Lake (Lake) were separately solubilized in 20 mL of TSB medium. Each medium (Volcano and Lake soil media) was centrifuged for 10 min at 9000 RMP and the supernatants were used as the growth media for the growth curves assays. Statistical differences were assessed by Student’s t test p < 0.05 or Mann–Whitney test p < 0.05 (GraphPad Prism 4).

### Neighbor-joining analysis

*Microbacterium* sp. CGR1 and CGR2 16 S rDNA sequences were obtained from NCBI under accession codes MK110962.1 and KU714726.1, respectively. The *Microbacterium* sp. CGR1 and CGR2 strains are available under the accession codes RGM2230 and RGM2255 from Colección Chilena de Recursos Genéticos Microbianos—INIA (RGM, Chillán, Chile), respectively.

For the construction of the Neighbor-joining tree, we analyzed 109 sequences corresponding to the best scored representatives of every named *Microbacterium* species of length longer than 1400 bp present in SILVA database (November 2018). These sequences were aligned using the MAFFT v7.407 software^20,21^, with the default algorithm (FFT-NS-2) and subsequently trimmed to 1315 bp of aligned length and realigned using the G-INS-I algorithm. MEGA-X v10.0.5 software^22^ was used to build a distance tree based on the neighbor-joining method under the Kimura-2-parameters substitution model^23^. Bootstrap analysis (1,000 pseudo-replicates) was used to evaluate statistical nodal support. The tree was visualized and annotated using the online tool Interactive Tree of Life v4.2.3^24^.

### Genome sequencing and sequence information

Genome sequence from *Microbacterium* sp. CGR1 was obtained from the GenBank BioProject PRJNA291433^5^. Genome sequencing of CGR2 strain was performed using Illumina GAIIx sequencing technology (Macrogen, Seoul, Korea). A shotgun library with a total of 4,169,378 paired end reads of 300 bp was generated (1,249 Mb of total data, 315x raw coverage). De novo assembly was conducted under quality-filtered reads using CLC Genomics Workbench version 8.5.1 with length and similarity cutoffs of 80% and 90%, respectively, resulting in four contigs with an N50 size of 3,676,265 and coverage of 238×. *Microbacterium* sp. CGR2 was annotated using the NCBI Prokaryotic Genome Annotation Pipeline released on 2013, version 4.6^25^ and approved on October 10th, 2018. Gene mining and genomic contexts were visualized using RAST server^26^.

### Genome annotation and Reconstruction of metabolic networks

Identification of coding regions, annotation and proteome comparisons were performed using previously described protocols. Briefly, a pipeline for prediction of coding regions and annotation was executed for the two strains. Using tRNAscan-SE^27^, we identified tRNAs over the draft genomes. Then, in order to find coding sequences, we used glimmer 3.02^28^. The functional annotation of coding sequences was performed using Blast comparison to several databases including NCBI Non Redundant, SWISSPROT, OMNIOME, KEGG, COG and InterproScan using PROSITE, Pfam, ProDom and SMART. Two GEMs were reconstructed for *Microbacterium* sp. CGR1 and *Microbacterium* sp. CGR2, namely Mcgr1 and Mcgr2. In order to minimize random biases from the process, both reconstructions were done following the same steps, which included: (1) Generation of draft models, (2) Generation of a biomass equation, (3) Gap filling, and (4) Test models. These steps were implemented using the following platforms: Aureme^29^, Pathway Tools^30^ and, COBRA (Matlab)^31^, as explained below.

First, draft models were generated for both *Microbacteria*, using the softwares Pantograph from AuReMe^32^ and PathoLogic from Pathway Tools^33^. Pantograph is based on orthology and GEMs available for *Mycobacterium tuberculosis* and *Streptomyces coelicolor* were used as templates^34,35^, as they are members of *Actinobacteria* and their GEMs have been extensively curated (comparison between all models are shown in Supplementary Fig. 1). In order to include reactions that could have been overlooked in these initial drafts, two more drafts were generated using Pathologic (PathwayTools), which predicts metabolic pathways based on annotated genomes, thus helping to confirm already selected reactions, and identifying new ones. The results from both approaches were manually merged, ensuring that the same metabolic pathways were evaluated in both reconstructions. Second, a biomass equation was generated to represent cell growth for each of these microorganisms. This biomass equation, identical for each *Microbacterium*, was generated utilizing biomass from GEMs of *Escherichia coli* and *Streptomyces coelicolor* as templates, including only components present in both, and adding additional organism-specific molecules from genome information (full list in Supplementary Table 1).

Then, the models were used to test the feasibility to produce biomass, and a manual process was implemented to fill gaps. This consisted of evaluating reactions present in the template model (*S. coelicolor*) that could account for the synthesis of biomass, considering whether reactions were still essential if individual metabolites were added to the media, and removing those reactions that lacked genome support and could be substituted for single metabolite additions. With this process, we identified that both *Microbacterium* lacked 11 reactions for the biosynthesis of biotin, and hence, rather than adding those reactions, biotin was added to the media considering that other organism (such as plant or fungi) are able to provide it to the environment^36,37^. Transport reactions were also added from template models. Both models included chemical equations consistent with those presented in the BiGG Models database^38^. Mass balance tests were performed in COBRA, to ensure that all internal reactions would be balanced. Finally, both models were tested to ensure that: (1) their reactions were elementally balanced, (2) they could generate biomass from defined media, and (3) they did not present futile cycles generating energy. Manual curation was done throughout the reconstruction process, as this allows evaluating the need to include different reactions, pathways, or biomass components.

### Carotenoid determination

For carotenoid extraction^39^, 40 mg of each bacterial or strain culture were pelleted. After supernatants were discarded, 400 μL of acid-washed glass beads (Sigma Aldrich) and 1 mL of hexane were added for cell disruption and carotenoid dissolution. Cells were disrupted at room temperature in a BeadBug6 cell homogenizer (Benchmark Scientific) using a program consisting in 4 cycles of 90 seconds of disruption at 3700 rpm followed by a 10-second rest. Cell lysate was then centrifuged and the liquid phase (hexane) was recovered and then evaporated by adding nitrogen on the surface of the liquid. After solvent evaporation, the solid residues in the bottom of the test tube were resuspended in 1 mL of acetone and vortexed until complete dissolution. If a white precipitate appeared, it was discarded by centrifugation. Finally, the homogenous acetone was transferred to a 1.5 mL Eppendorf tube and stored at −20 °C until HPLC analysis.

The carotenoid analysis was performed by LaChrom L-7000 chromatograph using Reversed-Phase High-Performance Liquid Chromatography (RP-HPLC) for detection^40^. Briefly, samples were pre-treated for injection by mixing 450 μL of sample with 50 μL of a 200 mg/L solution of β-apocarotenal, used as internal standard to correct the apocarotenal injection error (retention time tR~6.1 min). After adding the internal standard, 50 μL of sample were injected in duplicate into a LiChrospher® 100, RP C18 (5 μm) column, using Acetonitrile:Isopropanol:Methanol (85:10:5) as a mobile phase. Carotenoids were detected by measuring the optical density of the eluent of the column at 453 nm using a diode array detector (DAD). Chromatograms from the samples were compared with those of known standards to determine the presence of different carotenoids: lycopene (tR ~ 11 min), δ-carotene (tR ~ 13,6 min), ε-carotene (tR ~ 16.5 min) and β-carotene (tR ~ 19.7 min). All standards were purchased from Sigma-Aldrich.

## Results and Discussion

### Environmental adaptation assays

**Figure 1:**
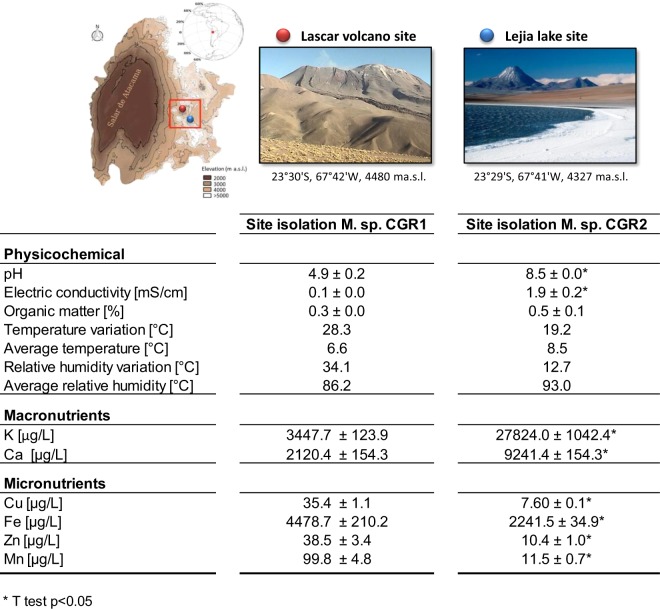
Geographical location of sampling sites where *Microbacterium sp*. CGR1 and CGR2 were isolated and characterization of their respective physicochemical environments. The two points denote the specific locations within the Atacama Desert corresponding to the Lascar Volcano and Lejia Lake sites. The table describes the contrasting physicochemical conditions.

The Atacama Desert is the oldest and driest desert on Earth. In recent years, it has become one of the principal areas for the study of microbial diversity, biogeochemistry and natural products potential^19,41^. Based on the particular soil scenario present in the Atacama Desert, two Gram-positive, rod-shaped and yellow-pigmented *Microbacterium* strains (CGR1 and CGR2) were isolated from soil samples collected from the Andes mountain range in the central region of the Atacama Desert (Fig. 1).

CGR1 was obtained from an acidic site with low salinity (pH 4.9; 0.1 mS/cm), close to the Lascar volcano, while CGR2 was isolated from an alkaline site with high electrical conductivity (pH 8.5; 2.0 mS/cm) located near Lejía lake. Analysis of available elements showed that the Lake site had a significant higher content of Ca, Mn and K nutrients, supporting its characterization as a salt lake. In contrast, Cu, Fe and Zn were higher at the Volcano site. A similar scenario has been observed in acid mine drainage environments, where the low pH facilitates the solubilization of heavy metals.

**Figure 2:**
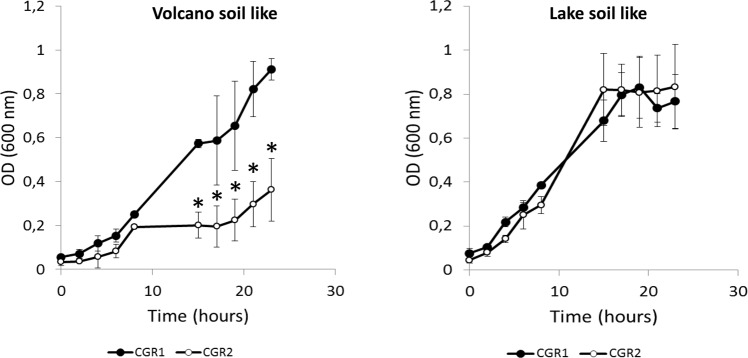
*Microbacterium sp*. CGR1 and CGR2 proliferation curves growing in volcano and lake soil media. Asterisk = significant differences between CCGR1 and CGR2 growing at the same condition and same time. Error bars = standard deviation (SD) values. (Mann–Whitney test, p < 0.05).

Thus, CGR1 and CGR2 were isolated from soils with highly contrasting physicochemical environmental features. In order to test if both bacteria are able to reciprocally growth in each other’s habitat, we generated two soil-like culture media (Volcano and Lake) to emulate the physicochemical variables present in the isolation sites (for details see Materials and Methods). Figure 2 describes the biomass concentration of both *Microbacterium* isolates growing in each of the soil-like media. The results showed that CGR1 grew under the Volcano and Lake media in a similar way. On the other hand, the emulated conditions of the Volcano soil reduced the proliferation of CGR2. As mentioned before, the main differences between both sites, and hence between both media, were the pH and salt concentrations (conductivity). In terms of pH, the Volcano soil media had a final concentration of protons equal to 1E-5, more than four orders of magnitude higher than the concentration of the Lake medium (pH = 9). In general, pH is one of the principal variables highly correlated with soil microbial diversity at the continental scale^42^, across artificial pH gradients^43^, in highly managed urban systems and in natural pH gradients^19^. As observed for other environmental factors, the peak of diversity has been correlated with near-neutral or optimal conditions, showing a trend to decrease towards more acidic and alkaline (extreme) conditions^42,44^.

Considering salts, the Lake medium had at least 10 times greater concentration of elements (such as K, Mg, Ca, S and Na) than the Volcano medium. Salinity has also been known as a factor that influences the microbial diversity of soil^45^. Bacteria that live in saline environments, especially when they are hypersaline, must overcome the essential problem of maintaining a balance in intra and extracellular water content, and thus avoiding cellular dehydration (Ventosa *et al*., 1998). For this reason, only osmotolerant species are able to inhabit high saline conditions, leading to a decrease in soil microbial diversity in these environments^46^.

### Phylogenetic analysis

The genus *Microbacterium* was first proposed by Orla-Jensen in 1919^47^, and was afterwards revised by Collins *et al*. (1983) and by Takeuchi & Hatano (1998a) in order to include the unification of *Microbacterium* with its closely related genus *Aureobacterium*^48,49^. At present (November 2018), the genus comprises 109 species (http://www.bacterio.net/microbacterium.html), which occupy a very wide and diverse environmental distribution, including soil^50^, water^51^, plants^52^, rhizosphere^53^ and human clinical specimens^48,54^.

**Figure 3:**
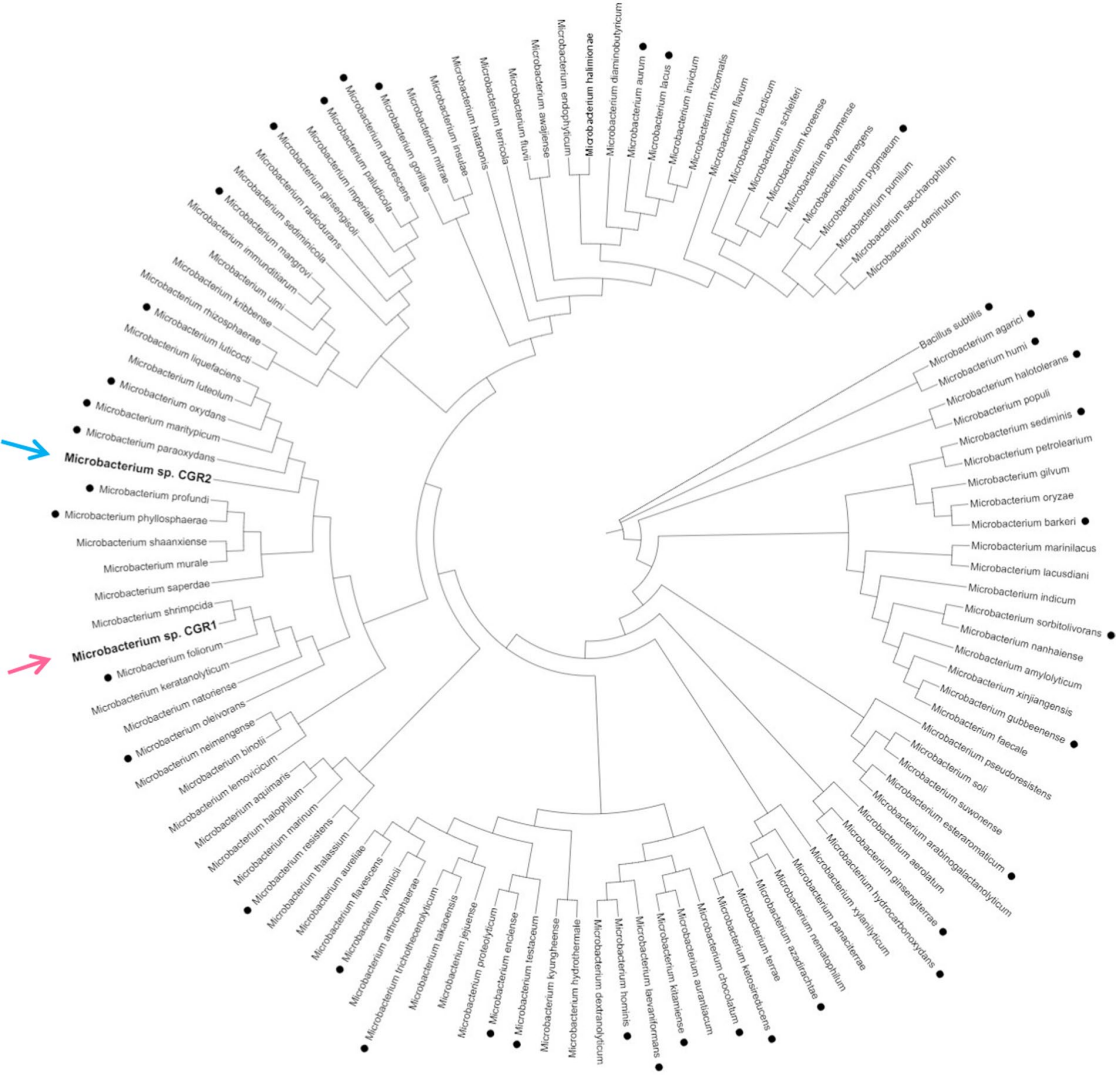
Phylogenetic tree (Neighbor-joining) derived from the analysis of 16 S rDNA sequences of *Microbacterium sp*. A total of 89 *Microbacterium* species were included in the tree. Red and blue arrows indicate the positions of *Microbacterium* sp. CGR1 and CGR2, respectively. Black dots indicate species with genome sequence available.

A phylogenetic analysis of both *Microbacterium* isolates from the Atacama Desert was performed, in which the 16 S rRNA sequences of CGR1 and CGR2 strains were compared with the 16 S rRNA sequences from 109 *Microbacterium* species (Fig. 3). The results revealed that CGR1 strain belongs to the same clade as *M. shrimpcida*, while CGR2 clusters together with five other species of *Microbacterium*. Therefore, despite the fact that both strains were isolated from neighboring sites, they appear to have different evolutionary histories and are likely to have arrived in the area independently. Furthermore, the capacity to survive in the markedly dissimilar Volcano and Lake soil environments, suggests the presence of molecular determinants which may relate to particular and/or general metabolic adjustments which are divergent between both *Microbacterium* strains.

### Genome-scale metabolic models: general features

Considering that these two strains were isolated from sites in the Atacama Desert with contrasting soil features, the identification of common and unique molecular determinants (genes, proteins, enzymes, pathways, etc.) could be useful to assess whether metabolic modifications can be linked to adaptations to their particular ecological constraints. For this reason, the global metabolic capabilities of both *Microbacterium* isolates in this study were assessed using their GEMs, and were named Mcgr1 and Mcgr2.

**Figure 4:**
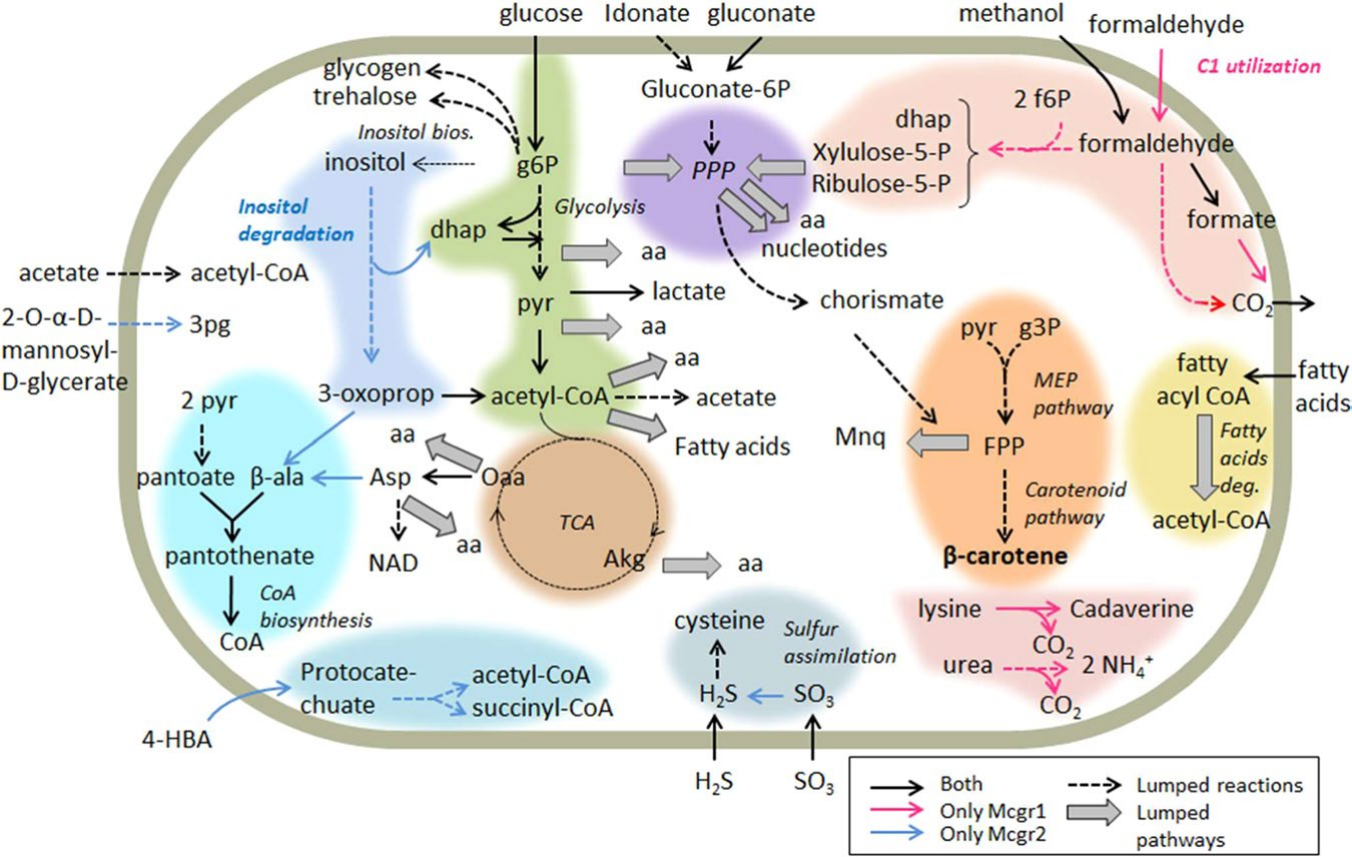
Integrative global metabolic model of *Microbacterium sp*. CGR1 and CGR2. The cellular model describes metabolic interconnections between different pathways related to basal metabolism. Colored and shaped arrows describe particular and common pathways and reactions between both *Microbacterium* GEMs and metabolites exchanged with the environment.

The Mcgr1 and Mcgr2 models contain 632 and 648 genes, which correspond to 19% and 17% of the total genes in each genome, respectively. These values are an expected and sufficient level of representation as they fall within the range of other GEMs (for example, the GEM for *Streptomyces coelicolor* iMK1208 contains 15% of genes^35^). The Mcgr1 and Mcgr2 models contain 1168 and 1172 reactions, respectively, from which 760 and 764 correspond to metabolic reactions. The similarity of the metabolic capabilities in core pathways is reflected by the high percentage of shared metabolic reactions (735, corresponding to 97% and 96% of Mcgr1 and Mcgr2, respectively). These similarities are also reflected by the number of shared metabolites among the two models (98% for both). Table 1 contains the metabolic characteristics of the genomes for CGR1 and CGR2, and their respective GEMs. The two GEMs were used to assess the metabolic characteristics with respect to substrate utilization and handling of stresses present in the desert. Figure 4 summarizes the shared and unique basal metabolism pathways in the Mcgr1 and Mcgr2 models. Overall, the convergence of metabolic properties between both strains allowed the identification of metabolic pathways and general metabolic strategies that could explain their survival in this extreme environment.

**Table 1:** General features of *Microbacterium* CGR1 and CGR2 metabolic models.

Metabolic Models	Mcgr1	Mcgr2
Genome size (MM pb)	3,63	3,68
Total protein coding genes in genome	3299	3908
Genes included	632 (19%)	648 (17%)
Reactions	1168	1172
Export and sink	186	186
Transport	221	221
Metabolic	760	764
Shared met. reactions	735	
Not shared met. reactions	25	29
Biomass	1	1
Metabolites	904	897
Unique metabolites	728	721
Shared metabolites	882	882
Not shared metabolites	22	15
Reactions with genes	816 (83%)	812 (82%)

**Figure 5:**
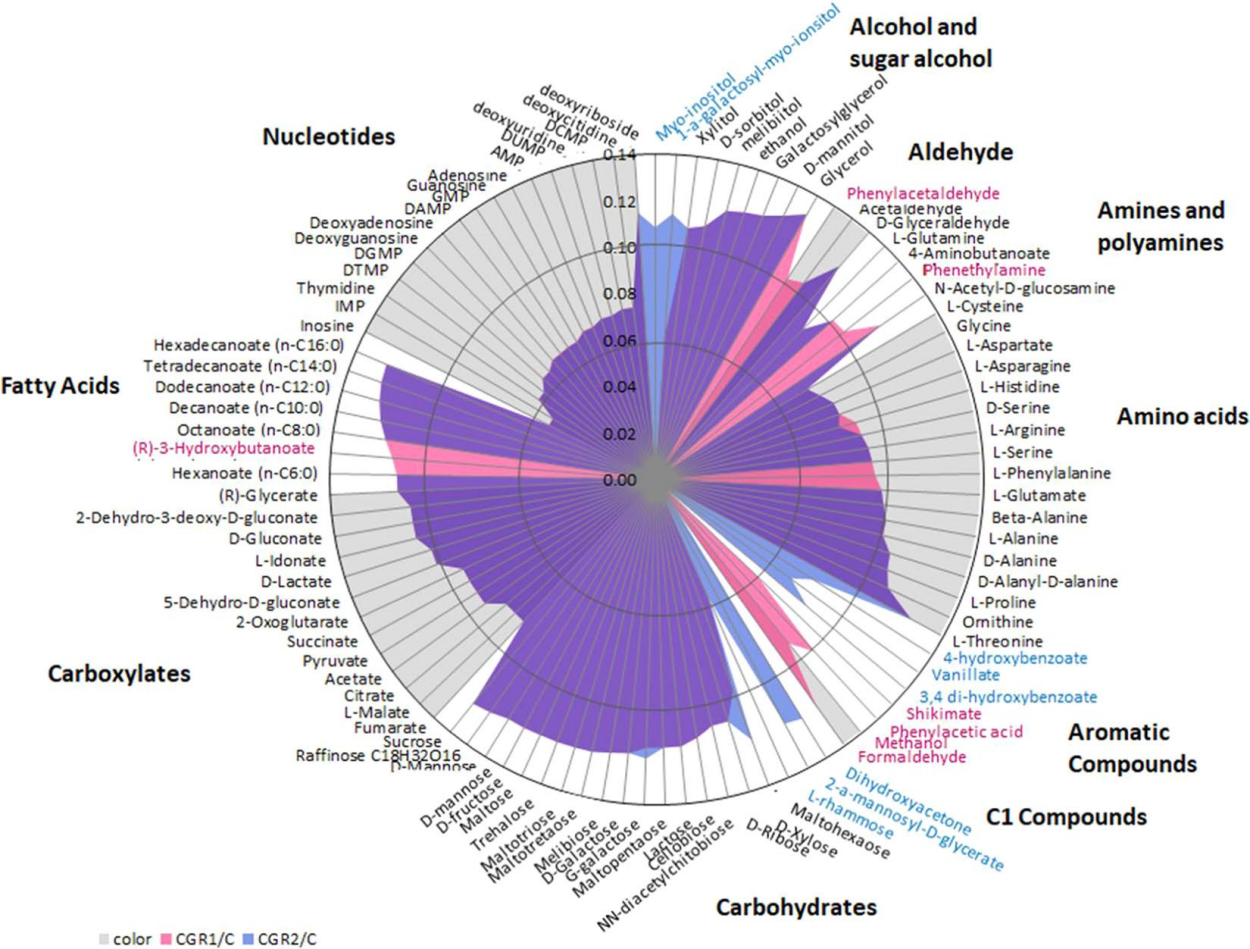
*In silico* analyses of potential substrates used by *Microbacterium* sp CGR1 and CGR2. The circle graph shows the predicted maximum growth rates (in 1/h) for each substrate shown. Color key: Pink for CGR1, blue for CGR2 and purple for both strains. Color key for substrate names indicates presence of such substrate in the model: pink for CGR1, blue for CGR2 and black for both. Note that presence does not imply carbon/energy source, as it is in the case of phenylalanine. Grey and white background are intended to separate substrates by families as follows: Grey for: aldehydes, amino acids, C1 compounds, carboxylates and nucleotides; white for alcohol and sugar alcohol, amines and polyamines, aromatic compounds, carbohydrates, and fatty acids.

*Actinobacteria* are known for their role in degrading and recycling diverse compounds, mainly sugars^55^, from their environments. Hence, our models were utilized to evaluate *in silico* the employment of a variety of potential carbon substrates. Our results indicate that both *Microbacterium* strains were highly versatile in the utilization of carbohydrates, carboxylates, amino acids and nucleotides. To a lesser extent, they can both utilize fatty acids, alcohols and sugar alcohols; Fig. 5 shows the result for 100 potential substrates used by the strains. Substrates were classified as alcohol and sugar alcohols, aldehydes, amines and polyamides, amino acids, aromatic compounds, C1 compounds, carbohydrates, carboxylates, fatty acids, and nucleotides.

Despite many similarities, the two *Microbacterium* strains differ in the utilization of several compounds, including phenyl, C1 and aromatic compounds, the latter two with ecological relevance. In the specific case of phenylalanine, both models have the ability to utilize it, however only Mcgr1 contains a reaction leading to its utilization as carbon/energy source resulting in growth. The predicted metabolic utilization of phenyl compounds by Mcgr1, and benzoate compounds by Mcgr2 does not seems to be connected. Based on the predicted metabolic models for both *Microbacterium*, phenyl-compounds in model mcgr1 are predicted to be utilized by pathways L-phenylalanine degradation II (anaerobic) followed by phenylacetate degradation I (aerobic). On the other hand, benzoate compounds are predicted to be metabolized into protocatechuate, and from there utilize pathways protocatechuate degradation II (ortho-cleavage pathway) and 3-oxoadipate degradation. C1 compounds include formaldehyde and methanol. Interestingly, Mcgr1 has three pathways to utilize C1 carbon sources, while Mcgr2 has none, indicating a robust system to deal with these compounds in Mcgr1. This robustness may also ensure that formaldehyde, a toxic metabolite, is rapidly recycled^56^. Since Mcgr1 was isolated from the Lascar volcano, it is possible that the proximity to the volcano may elevate the availability of methanol and formaldehyde in the environment. For example, Lynch and collaborators identified formaldehyde and other C1 gases present in another volcano in the Atacama Desert, together with biodegradation pathways in bacteria from that area^4^.

Aromatic compounds are another family of substrates belonging to pathways that show differences between Mcgr1 and Mcgr2, as only Mcgr2 can utilize 4-hydroxybenzoate (4-HBA), vanillate and 3,4-hydroxybenzoate (protocatechuate), the latter being also an intermediate compound in the degradation of the former two. These components are considered aromatic pollutants, and their bacterial degradation has been the focus of previous studies^57^, with more particular emphasis in the bacterium *Pseudomonas putida*^58^. Other *Microbacteria* in different locations have been reported to degrade protocatechuate. According to the pathway database available in Pathway Tools^30^, out of the 48 *Microbacteria* included, 39 possess protocatechuate degradation pathways. In contrast, only one model in the BiGG database contains this compound (*P. putidas*, iJN746)^38^, indicating underrepresentation in constructed models.

Another difference between Mcgr1 and Mcgr2 was that only Mcgr2 was capable of degrading inositol. Since this compound is an osmoprotectant for several bacteria and plants^59^, we theorize that Mcgr2 has adapted to utilize this component produced by other organisms. Mcgr2 was isolated from a salt lake, where it is expected that plants and microorganisms could produce inositol as an osmoprotectant.

Microbacterium species have been previous classify as putative methylotrophic bacteria^60^. In order to improve the understanding of this point, we compared the metabolism of both *Microbacterium* strains against the GEMs of different species with C1 metabolism, which included the models from *Methylococcus capsulatus*, *Bacillus subtilis* and *Pseudomonas putida*^61–63^. For this, we identify the presence of 16 enzymes involved in 4 pathways related to C1 metabolism: formaldehyde assimilation II (assimilatory RuMP Cycle), formaldehyde oxidation I (dissimilatory RUMP cycle), formaldehyde oxidation IV and methanol oxidation in the genomes of all five species. We found out that both *Microbacterium* are the only two species containing all these enzymes, supporting the idea that CGR1 and CGR2 belong to the group of methylotrophic bacteria. Interestingly, by calculating the connectivity of metabolites, we found out that formate, formaldehyde, methanol and CO_2_ have a lower connectivity in *Microbacterium* than the other species. This result suggests that the C1 metabolism in the *Microbacterium* CGR1 and CGR2 presents a high level of specificity, conferring to the systems the a low level of connectivity inside the network^64^, a characteristic observed in specialized species that live in particular niches, such as *Geobacter sulfurreducens*, a soil sulphur-reducing proteobacterium^65^.

A second analyses was performed comparing the metabolism of both *Microbacterium* against *Chromohalobacter salexigens* (formerly *Halomonas elongata*)^66^, which is a halophilic extremophile commonly used to study osmoadaptation and the methanotrophs bacteria *Methylomicrobium buryatense* (adapted to metabolize C1 compounds)^67^. All models contain a similar number of enzymes, reactions and metabolites. In particular, one of the main mechanisms of the latter to cope with the saline environment is the production of osmoprotectants such as ectoine and hydroxyectoine, both metabolites able to be synthesized by *Chromohalobacter salexigens* and both *Microbacterium* strains. The model for the methanotrophs bacteria *Methylomicrobium buryatense*, utilizes the RuMP cycle to fixate C1 just like *Microbacterium* Mcgr1. This pathway is a highly efficient route for the assimilation of reduced one-carbon compounds (Formaldehyde). Considering that CGR1 soil contains higher metal concentration (Fig. 1), the possibility to use an alternative carbon source gives to CGR1 an important advantage to produce energy (ATP), a crucial metabolite used for metal efflux ATPase, also with interesting biotechnological applications^68^.

### Metabolic response to desert-specific stresses

Considering the physicochemical differences between the isolation sites of both *Microbacterium* strains, mainly salt concentration and pH, we evaluated their metabolic support for osmotic and acidic/alkaline stresses.

Microbial mechanisms to deal with osmotic stress have been previously described and can be summarized in two main categories: K + transport and biosynthesis (or transport) of osmolytes. Some osmolytes that have been reported for bacteria include glycine-betaine, carnitine, proline, trehalose, ectoine and glutamate^69^. We evaluated, *in silico*, the feasibility of Mcgr1 and Mcgr2 to synthesize these products and found that both *Microbacterium* strains have the potential to synthesize high levels of proline, trehalose and glutamate (Supplementary Fig. 1; the full list of osmolytes biosynthesized by Mcgr1 and Mcgr2 are listed in Supplementary Table 2). Additionally, as mentioned above, Mcgr1 and Mcgr2 are capable of synthesizing inositol, which has been identified as an osmolyte in plants^70^. Thus, we hypothesized that inositol may offer osmotic protection to the *Microbacterium* genus^59,69^. Interestingly, biosynthesis of inositol is common between eukaryotes, but not among bacteria. Besides, in prokaryotes, inositol also acts as a precursor of cell membrane molecules^71^. Overall, the *in silico* simulation showed that both Mcgr1 and Mcgr2 have the capability of synthesizing several osmolytes that could allow these bacteria to endure osmotic stress, although their actual roles should be verified experimentally.

*In silico* analysis showed that both *Microbacterium* isolates are equally capable of adjusting to the addition or removal of protons (i.e., same cell growth prediction for each model). Hence, we hypothesized that their different capacity to deal with acidic conditions could be explained by system-level properties, such as metabolite connectivity (e.g., the same metabolite participating in more than one reaction or pathway)^72^. Table 2 summarizes all metabolites with connectivity differences of 3 and higher (full list in Supplementary Table 3). Interestingly, CO_2_ was one of the top metabolites, with 5 degrees of difference in connectivity, with higher differences in Mcgr1 compared to Mcgr2. Since CO_2_ is often involved in decarboxylation reactions, and previous studies have shown that decarboxylation reactions could participate in mechanism to handle acidic stress by incorporating protons^73^, we looked at these reactions in more detail in the models. We found that the gene AKG07_04960 is only present in the Mcgr1 genome. It encodes for a decarboxylase of the amino acids lysine, ornithine and arginine, which, interestingly, have been shown to play a role in the response to acidic stress and survival in other bacteria^74^.

**Table 2:** Metabolites connectivity analysis.

Metabolites	Total connectivity		
	Mcgr1	Mcgr2	Difference
D-Glucose 1-phosphate	12	6	6
CO_2_	65	60	5
NAD	77	73	4
NADH	71	67	4
Pi	138	135	3
3,4-Dihydroxybenzoate	1	4	−3
Adenosine 5′-phosphosulfate	0	3	−3
ADP	140	144	−4
H+	527	531	−4
ATP	196	202	−6
H_2_O	292	298	−6

Differences were calculated using Mcgr1 as reference.

Another mechanism that has been described in the literature as an important player in the handling of alkaline and acidic stress is the direct exchange of protons with the external environment through proton pump mechanisms ^73,75,76^. In this respect, both *Microbacterium* strains have a gene, Sodium/Proton antiporter (AKG07_14825 in Mcgr1, and Microbacterium_sp_CGR2-contig1_1373 in Mcgr2), predicted to be pH-dependent that could be involved in maintaining proper homeostasis under alkaline conditions, as they both have the capability to grow in extreme alkaline conditions (see Fig. 1**)**. Therefore, we suggest that the connectivity of decarboxylation reactions and proton exchange represent a common metabolic strategy that could explain the presence of both strains in this extreme environment. At the same time, variations in the magnitude of this connectivity could affect their pH tolerance range.

We also compared the *Microbacterium* GEMs against the extremely halophilic bacterium *Salinibacter ruber* in order to find specific mechanisms of adaptation to high concentrations of salt^77^. *S. ruber* is characterized by having different osmotic resistance proteins also conserved in Archaea species, such as light-driven proton and chloride pumps, none of them encoded in CGR1 or CGR2 genomes. While the *Microbacterium* CGR2 lives in a salt environment (Lejia Lake), both not need high concentration of salt to growth, unlike *S. ruber*, which optimal growth performance requires a 20–30% of salt in the media. In addition, *S. ruber* are able to produce an unusual carotenoid called salinixanthin, a compound used for transfer energy to light-driven proton pumps. Interestingly, according to the GEMs, both *Microbacterium* are able to produce carotenoids, however, the absence of light-driven proton pumps in these bacteria suggest that these molecules are not directly related to salt resistant.

### Metabolic pathway and production of carotenoid

Another relevant stressor in the Atacama Desert is the high UV radiation, which could severely impair growth of certain bacteria, while not having a significant impact in others due to the presence of protective mechanisms^78^. Microbial mechanisms to deal with radiation stress include DNA repair mechanisms and genetic expression of photoprotection molecules^79,80^. One group of photoprotection molecules are carotenoids, long chain molecules that provide pigmentation to many organisms and can absorb UV wave length that might otherwise damage cellular structures^81^. Multiple studies have positively correlated carotenoid pigmentation with UV resistance in bacteria^82,83^. It has also been shown that pigmentation by itself does not explain the overall bacterial response to UV radiation in some populations^84^, a situation that we believe is explained by the presence of different, and possibly complimentary, protective mechanisms.

**Figure 6:**
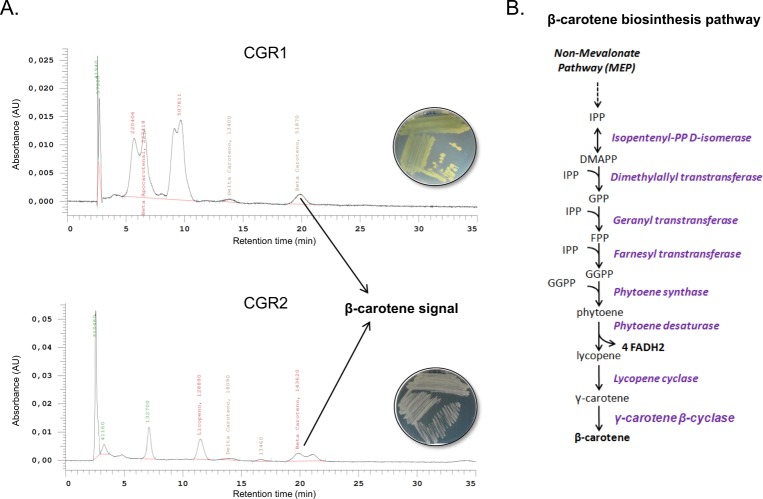
Identification of carotenoid identification compounds produced by *Microbacterium sp*. CGR1 and CGR2. (**A**) HPLC chromatogram of CGR1 and CGR2 extract containing different pigmented molecules. Plates show the color of both *Microbacterium* strains under study. (**C**) Conserved biosynthesis pathways for β-carotene in CGR1 and CGR2.

*Microbacterium* species have been widely used in biotechnological applications; in particular their production of carotenoids has be used for diet supplements, food colorants, animal feed and nutraceuticals^85^. Interestingly, both Mcgr1 and Mcgr2 exhibited pigmentation on petri plates, and they both possessed biosynthetic gene clusters for carotenes, which strongly suggest that they are producers of carotenes (Fig. 6A,B). To validate this prediction, we measured carotenoids by HPLC, finding carotenoid products on both *Microbacterium* strains, specifically β-carotene. Our orthology-based reconstruction system identified that both strains contained the biosynthetic pathway to synthesize the key precursor Isopentenyl pyrophosphate (IPP) in the non-mevalonate pathway (Fig. 6B). This was expected since this is the preferred pathway in bacteria for carotenoid production^86^. Since the biosynthesis of β-carotene and its benefits to human health have been the focus of intense research^87^, we decided to estimate the metabolic cost associated with this biosynthesis in the two desert *Microbacterium* species. Not surprisingly, *in silico* biosynthesis of β-carotene is among the most expensive possible products evaluated in this study, as the process includes several energy requiring reactions.

Synthesis of other metabolites could also contribute to UV radiation resistance in certain bacteria. For example, a recent study showed that synthesis of the osmolytes inositol and glycine-betaine became upregulated when cells were exposed to high UV radiation. This indicates that these compounds could have a double role in the *Microbacterium* isolates of this study in addition to their possible role as osmoprotectants^88^. Finally, the chromatogram from Fig. 6A indicated the accumulation of other carotenoids, such as lycopene (precursor of β-carotene), δ-carotene and other pigmented molecules, which open the opportunity for new studies in CGR1 and CGR2 and potential biotechnological applications.

## Conclusion

The repertoire of strategies that bacteria use to adapt to extreme environments can potentially be used to discover new pathways of interest for biotechnological approaches. In this context, the desert environment is especially attractive, since microorganism growing in challenging conditions, such as extremely low water and nutrient-poor soils, temperature oscillations, constant solar radiation, different salinity conditions and varying pH.

In this work, we presented the first two GEMs for *Microbacterium* species isolated from two contrasting sites in the Atacama Desert. The exhaustive exploration of both models showed a high conservation in basal metabolism, where apparently the internal connectivity of particular metabolites (e.g., CO_2_) may have provided one species with a higher capacity to tolerate a wide range of pH. This comparative global metabolic analysis supports the idea that only a fraction of the encoded proteins takes an active part in metabolic pathways and display strain-specific correlation patterns. These patterns are reflective of the functional differentiation of the two *Microbacterium* strains described in this work, organisms that co-occur in microbial communities located in spatially close soils.

In addition, analysis of secondary metabolites revealed that both *Microbacterium* strains encoded the complete pathways required to produce different carotenoids, which are interesting targets for further development. Finally, together with the GEM model of *Streptomyces leeuwenhoekii* C34^17^, *Microbacterium sp*. CGR1 and CGR2 models are the first curated GEMs from bacterial species isolated from desert environments, significantly expanding the current knowledge of global metabolism in extreme bacteria.

## Supplementary information

GEM Mcgr1.

GEM Mcgr2.

Supplementary Figures.

Supplementary Tables.
