# Supplementary material for: Genome-scale metabolic models of Microbacterium species isolated from a high altitude desert environment
Source: Sci Rep. 2020 Mar 27;10:5560. doi: 10.1038/s41598-020-62130-8 (PMC7101325; doi:10.1038/s41598-020-62130-8)
Supplement: Supplementary file 3 — Supplementary Figures. [file 41598_2020_62130_MOESM3_ESM.pdf]

Title
Genome-scale metabolic models of *Microbacterium* species isolated from a high altitude desert environment.

Authors
Dinka Mandakovic, Ángela Cintolesi, Jonathan Maldonado, Sebastián Mendoza, Méziane Aïte, Alexis Gaete, Francisco Saitua, Miguel Allende, Verónica Cambiazo, Anne Siegel, Alejandro Maass, Mauricio González and Mauricio Latorre

Supplementary Information

Supplementary Figure 1. Reaction comparison between the *Microbacterium* metabolic models and the templates *Mycobacterium tuberculosis* and *Streptomyces coelicolor*.

Supplementary Figure 2. Metabolic pathways involved in osmotic stress response identified in *Microbacterium sp.* CGR1 and CGR2.

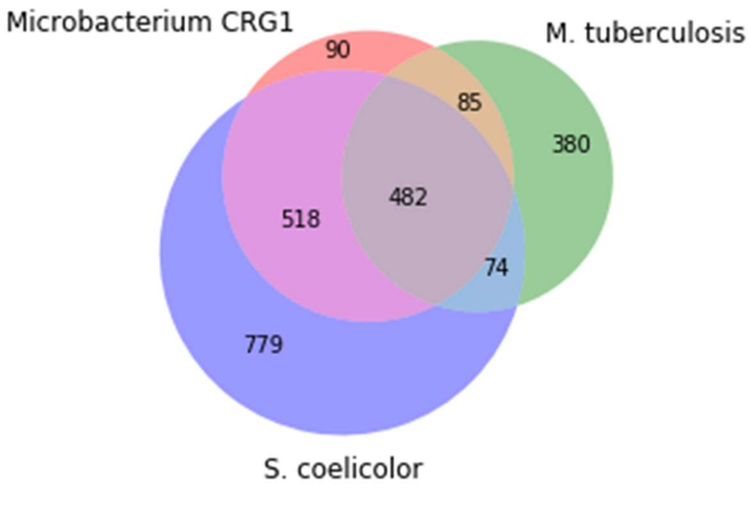

Supplementary Figure 1. Reaction comparison between the *Microbacterium* metabolic models and the templates *Mycobacterium tuberculosis* and *Streptomyces coelicolor*. Nearly to the 47% and 85% of the reactions of CRG1 and CRG2 are shared with *M. tuberculosis* and *S. coelicolor*, respectively.

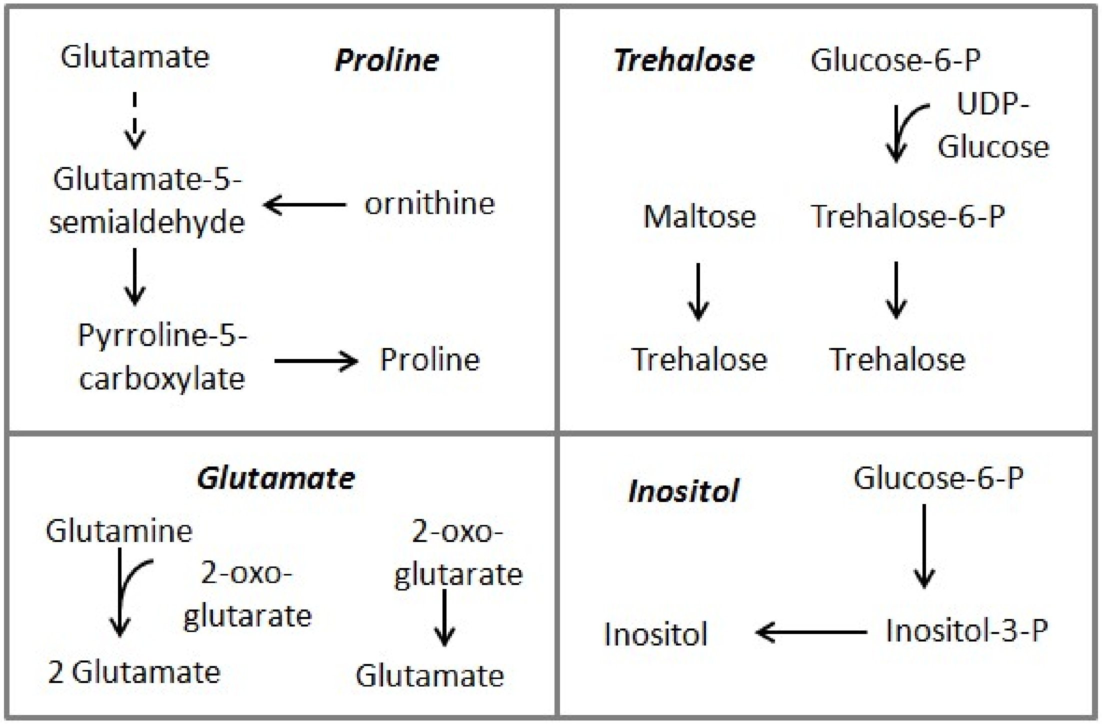

Supplementary Figure 2. Metabolic pathways involved in osmotic stress response identified in Microbacterium sp. CGR1 and CGR2.
